# Haemoglobin, anaemia, dementia and cognitive decline in the elderly, a systematic review

**DOI:** 10.1186/1471-2318-8-18

**Published:** 2008-08-08

**Authors:** Ruth Peters, Lisa Burch, James Warner, Nigel Beckett, Ruth Poulter, Christopher Bulpitt

**Affiliations:** 1 Experimental Medicine and Toxicology, Imperial College Faculty of Medicine, Hammersmith campus, Du Cane Road, London, W12 0NN, UK; 2 St Charles Hospital, Exmoor Street, London ,W10 6DZ, UK

## Abstract

**Background:**

Anaemia may increase risk of dementia or cognitive decline. There is also evidence that high haemoglobin levels increase risk of stroke, and consequently possible cognitive impairment. The elderly are more at risk of developing dementia and are also more likely to suffer from anaemia, although there is relatively little longitudinal literature addressing this association.

**Methods:**

To evaluate the evidence for any relationship between incident cognitive decline or dementia in the elderly and anaemia or haemoglobin level, we conducted a systematic review and meta-analyses of peer reviewed publications. Medline, Embase and PsychInfo were searched for English language publications between 1996 and 2006. Criteria for inclusion were longitudinal studies of subjects aged ≥65, with primary outcomes of incident dementia or cognitive decline. Other designs were excluded.

**Results:**

Three papers were identified and only two were able to be combined into a meta-analysis. The pooled hazard ratio for these two studies was 1.94 (95 percent confidence intervals of 1.32–2.87) showing a significantly increased risk of incident dementia with anaemia. It was not possible to investigate the effect of higher levels of haemoglobin.

**Conclusion:**

Anaemia is one factor to bear in mind when evaluating risk of incident dementia. However, there are few data available and the studies were methodologically varied so a cautionary note needs to be sounded and our primary recommendation is that further robust research be carried out.

## Background

Low and high levels of haemoglobin may indicate increased risk of morbidity beyond the immediate cause of haemoglobin abnormality. Higher haemoglobin levels and polycythemia are associated with circulatory disturbances and risk of cardiovascular events, mortality and stroke [1,2]. Low levels of haemoglobin and the presence of anaemia have been found to be risk factors for poor mobility, increased frailty and decreased executive function in women [3-5], a relationship with poorer outcome after sub arachnoid haemorrhage [6] decreased motor performance [7] and increased risk of death [8]. Furthermore, 5797 participants in the cardiovascular health study (participants aged 65 or over) showed a 'reverse J' shaped curve relationship between mortality and haemoglobin levels [9]. Both lower (< 13.7 gm/dl for women and < 12.6 gm/dl for men) and higher (> 14.1 gm/dl for women and > 15.6 gm/dl for men) haemoglobin levels conferred a significantly increased risk of death. In the higher category this was a statistically significant 17 percent increase [9].

In the Women's Health and Aging Study [WHAS] the reference value used was > 12 to < 15 and the reverse J shape was seen although only anaemia (not polycythaemia) was significantly associated with mortality [3-5]. Anaemia prevalence rates are reported at around 25 percent [10-12] (30% in men 17% in women) of individuals over the age of 85 when defined according to the WHO criteria [13] and the literature generally agrees that a fall in haemoglobin levels in the eighth decade of life occurs and that this may be part of normal ageing [14,15].

The elderly, particularly the very elderly, aged eighty and over, are the fastest growing part of the population worldwide [16], and are at high risk of dementia and related cognitive decline. If haemoglobin levels are related to either incident or prevalent dementia or cognitive decline, there may be a possibility of intervention to prevent or ameliorate onset of dementia; important both for potential sufferers, carers, and, in economic terms [17]. A previous review focusing on the elderly did not provide meta-analytic data, did not examine higher levels of haemoglobin and did not focus on dementia or cognitive decline [18]. Since that review, the literature has grown and the very elderly have become even more of an important group in society, we have therefore performed a systematic review, focused on dementia and cognitive decline.

Objective: to review the evidence for a relationship between haemoglobin levels and cognitive decline/dementia in the elderly via a systematic review; and to evaluate the strength of the findings taking into account the methodological differences in constituent studies.

### Hypotheses

#### Haemoglobin as a risk factor

1: That anaemia (labelled as such in the literature) or as defined by the WHO as low levels of haemoglobin (< 12 gm/dl women and < 13 gm/dl men) will be predictive of lower cognitive function and or dementia.

2: That high haemoglobin (> 14.1 gm/dl women and > 15.6 gm/dl men) will be predictive of lower cognitive function and or dementia.

## Methods

**Figure 1:**
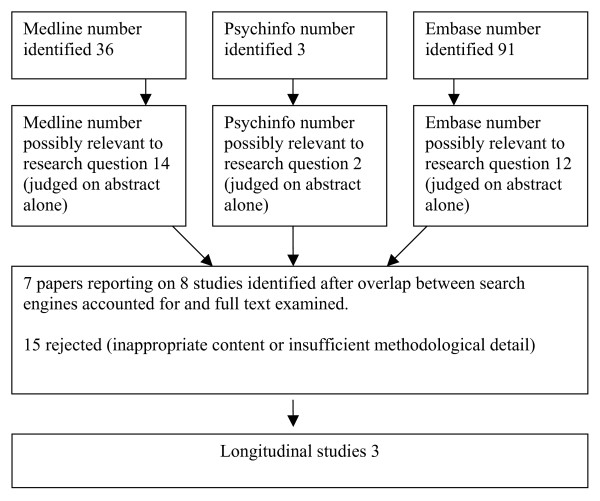
Showing results of systematic review.

In order to evaluate the evidence that was available in the scientific literature, a review was carried out using systematic review methodology in order to examine the literature in the most thorough and unbiased manner. Search terms 'anaemia' or 'anemia' or 'haemoglobin' or 'hemoglobin' and 'dementia' or vascular dementia' or 'multi infarct dementia' or Alzheimer's disease' or 'cognitive impairment' or 'cognitive decline' were used as keywords and the databases Medline, Embase and Psychinfo were searched for English language publications relating to human populations and occurring between 1996 and March 2006. The last 10 years were chosen as research methodology has evolved and computing power changed rapidly prior to this time. When available, standard search categories were also used as matched the above terms. All searches were limited to subjects aged 65 and over. See figure 1 for details. Two researchers appraised all abstracts independently; any discrepancies in decisions were discussed to achieve a unanimous choice of articles.

Studies were ranked on a methodological basis with randomised studies first, followed by case control and epidemiological population studies, and quality assessed using key factors including appropriate design, recruitment, analysis and provision of suitable information relating to key aspects of the study. Case studies, letters, consensus opinion from conferences and expert opinions or editorials were not included. In order to aid investigation of causality only longitudinal studies were included. Studies reporting results that could be used in a meta-analysis were analysed accordingly and tests for heterogeneity carried out.

## Results

From an initial 21 papers identified as relevant based on abstract alone only three remained so after full evaluation. Study quality varied and there were no randomised controlled trials or intervention studies of any sort. Methodological details and meta-analytical results are discussed below. Table 1 details the studies included.

**Table 1:** Characteristics of the three longitudinal studies

Author	Population Baseline numbers Descriptives Length of follow up [fu]	Type of study, main outcome	Measurement of cognition/dementia	Measurement of haemoglobin [hb]/anaemia	Adjusted for:	Relationship with haemoglobin [hb]/anaemia	Statistics if available. OR = Odds Ratio, RR = Relative Risk, HR = Hazard Ratio, (numbers in brackets are 95% confidence intervals) In order to assess the most conservative finding, results used are those from the adjusted analyses unless otherwise stated.
Atti et al [19]	Kungsholmen project, Sweden 1435 people without dementia at baseline Aged 75–95 years Mean fu 3.4(0.5 SD) years	Cohort Dementia	Clinical exam, review by specialist using DSM-III-R – dementia information on those who died in follow up from death certificates and medical records	WHO criteria 130 g/L men, 120 g/L women Plus: Lowest 25^th ^percentile 135 d/L men & 129 g/L women and lowest 5^th ^percentile 117 g/L & 116 g/L women	In those with MMSE >= 26: Age, sex, education, history of hypertension, diabetes, cerebrovascular disease, heart failure, chronic coronary disease, COPD, hypothyroidism, chronic renal failure, high white blood cells, high blood sedimentation rate, low albumin, low BMI.	Anaemia increases risk of dementia in those with MMSE >= 26 Low Hb-below 130 g/L men & 120 g/L women increases risk of dementia	In addition in subjects with MMSE >= 26.... With Hb < 25^th ^percentile HR 1.2 (0.7–2.0) With Hb WHO criteria HR 2.0 (1.0–3.8) With Hb < 5^th ^percentile HR 2.2 (1.0–4.9)
Atkinson et al [21]	Women's health & ageing study 1, USA 558 women with MMSE > 24 and walking speed > 0.4 m/s. Mean age of whole study = 78 yrs(SD8.1) Fu 3 years	Cohort Cognitive decline (fall in MMSE to < 24)	MMSE	Hb(g/dl)	Age, race, smoking, education, no diseases, pulmonary disease, hb, baseline walking speed, baseline MMSE, baseline IADL, baseline ADL	Hb not significant association with cognitive decline	Cognitive decline OR 0.86 (0.60–1.21) Combined physical and cognitive decline OR 0.68 (0.47–0.98)
C.Mary Beard et al [20]	Minnesota 1. 302 incident cases of Alzheimer's Disease [AD] with matched controls. (255 pairs analysed) Fu: Hb from year preceding or year of dementia onset.	1. Retrospective case-control	1. AD identified from lists of diagnostic terms thought to include dementia – diagnostic criteria similar to DSM. controls matched by age and gender.	WHO criteria 130 g/L men, 120 g/L women. 1. Lowest measurement recorded in 2 yr window (onset + preceding yr) for cases and corresponding for controls.	1. age, gender	1. Anaemia increases risk of AD (not significant in men)	1.OR 188 (1.17–3.03) Men OR 1.81 (0.75–4.39) Women OR 1.96 (1.11–3.47)
	2. 618 people Aged 65 years+ Fu: 5.1 years (1 day-7.9 years)	2. Cohort Alzheimer's disease [AD]	2. Medical records reviewed by nurse abstractor and confirmed by author	2. A value below WHO criteria between 1985 & 1989		2.No association between anaemia and AD	2.SIR 0.98 (0.67–1.37) Men SIR 1.49 (0.79–2.56) Women SIR 0.79 (0.49–1.23)

### Results of included studies and meta-analysis

**For the hypothesis that anaemia or low haemoglobin was predictive for dementia or cognitive decline; two studies using the WHO criteria for anaemia found significant results**.

The population study the Kungsholmen project found a Hazard Ratio [HR] of 2.0 (95% CI 1.0–3.8) for incident dementia with anaemia [19]. The retrospective case control study from Beard et al [20] found a similar result for Alzheimer's disease which was significant only in women (Odds Ratio [OR] 1.96 (95%CI 1.11–3.47)) although men showed a similar but non-significant effect OR 1.81 (95% CI 0.75–4.39). An additional analysis from the Kungsholmen population found an even stronger relationship with haemoglobin values below 11.7 g/dl for men and 11.6 g/dl for women respectively (The fifth percentile from their population). HR 2.2 (95% CI 1.0–4.9). [19]. No other evidence relating to low haemoglobin concentrations other than those defined above was available.

**Figure 2:**
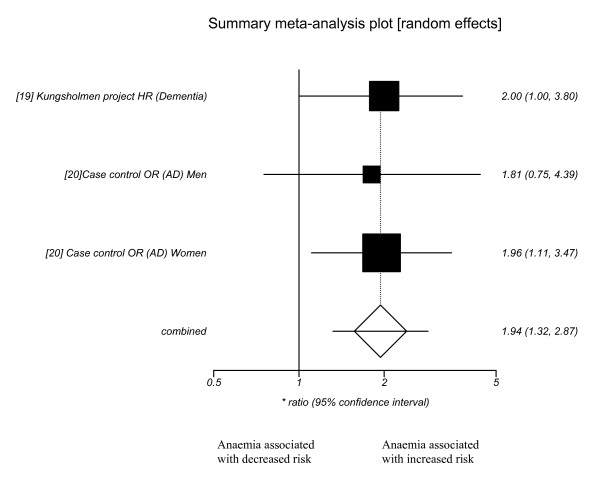
Forest plot for longitudinal studies; anaemia and risk of incident dementia.

It was only possible to combine two longitudinal papers in a meta-analysis to examine the relationship between anaemia and dementia (figure 2). The analysis of heterogeneity was non significant (p = 0.98). The pooled ratio was significant at 1.94 (95% CI 1.32–2.87). It was not feasible to include the cohort study from Beard et al in the analysis, as it reported only standardised incidence rates (a ratio of the incidence rate in the study population to that expected in a 'standard' population) and the source of the standard population data was unclear [20]. No analyses regarding haemoglobin concentration or cognitive decline were possible.

Higher haemoglobin gave rise to an odds ratio of 0.86 for incident cognitive decline in the WHAS study but this was non significant (95% CI 0.60–1.21) [21].

### Methodological considerations

#### Study design

Two of the longitudinal studies were population based cohort designs [19,21] with prevalent cases of dementia plus those with baseline MMSE below 20 and below 24 excluded respectively. The remaining longitudinal study reported on both a retrospective case control, (where haemoglobin values taken up to two years before incident dementia were used), and a cohort, (where all participants had newly recognized anaemia and were subsequently followed up) [20].

#### Study population

Longitudinal study populations were homogeneous and from northern Europe [19] and the United States [20,21] with age ranges or mean ages between 65 and 95 and therefore old enough to manifest dementia cases. Studies were relatively large with patient numbers ranging from 255 case control pairs [20] to 1435 followed for a mean of 3.4 years (Standard Deviation [SD]0.5) [19]. Follow up was less clear with one paper including a cohort follow up and a case control sub study of incident cases with retrospective haemoglobin measurements from the year of and the year preceding the onset of dementia [20]. Cohort follow up was wide ranging from one day to 7.9 years. This follow up included patients who had poorer cognition and who were not included in the analysis, there were 2417 person-years of follow up for the group that were analysed but the range was not clear [20]. Follow up in the study including only women was three years [21].

#### Outcome

There was some heterogeneity in the cognitive assessment outcome of the identified studies and standardised criteria were not always applied. Of the three studies, two assessed dementia and one cognitive decline. Only one, the Kungsholmen project, used the standardised Diagnostic and Statistical Manual of mental disorders [DSM] criteria, (the [DSM-III-R]) supplemented with information from clinical examination, death certificates and medical records [19]. Beard et al [20] reported on Alzheimer's disease but used an adapted form of diagnostic criteria, similar to the DSM. The WHAS [21] defined cognitive decline as a fall in MMSE to less than 24.

When measurement of haemoglobin or anaemia was considered two [19,20] used the WHO criteria for anaemia of less that 13 g/dl for men and less than 12 g/dl for women [15]. Atkinson et al reported using haemoglobin measures but no specific cut off values for anaemia. There was some discussion in the literature with regard to the utility of the WHO cut-off values and the Kungsholmen project also analysed their data using cut off values from the 25^th ^and 5 percentiles (13.5 g/dl men, 12.9 g/dl women and 11.7 g/dl men, 11.6 g/dl women respectively).

#### Variables adjusted for, and data from subjects not included or lost to follow up

Comprehensive adjustments for confounding variables were made only in the Kungsholmen population (Age, sex, education, history of hypertension, diabetes, cerebrovascular disease, heart failure, chronic coronary disease, COPD, hypothyroidism, chronic renal failure, high white blood cells, high blood sedimentation rate, low albumin, low BMI.)[19] and WHAS (Age, race, smoking, education, no diseases, pulmonary disease, haemoglobin, baseline walking speed, baseline MMSE, baseline IADL, baseline ADL) [21] studies.

Of the longitudinal studies only one took into account the effect of patients who died or left the study [19]. Death certificates were assessed to identify dementia.

## Discussion

The literature available to date in this area is limited and few data were available for analysis. Despite this, the available data are considered and presented here in order to give a robust review of findings to date. It is clear from this review that further research needs to be carried out in this area to strengthen the literature case and aid the ability to draw firm conclusions.

After the search and review process, three longitudinal studies were identified and considered in this review. The most robust one of which was the large population study, the Kungsholmen project [19]. The WHAS [21] was also of high quality and both found an increased risk of dementia or cognitive decline at later follow up with anaemia at baseline. The third paper reported on two studies and was weaker in that the length of follow up was varied for the cohort and unclear for the case control [20] the latter of which was significant only and supported the relationship between anaemia and dementia. It was only possible to combine the case control study from Beard et al and the Kungsholmen data in a meta-analytic way and the result was unsurprisingly significant given its constituent parts.

In reference to our hypotheses, the literature suggests that low haemoglobin or anaemia increases the risk of incident dementia or cognitive decline, although the available studies are few.

There were no data available that allowed examination of the hypothesis relating to high concentrations of haemoglobin.

It can be imagined that low haemoglobin or diagnosed anaemia could impact on future cognitive impairment, either directly by reducing blood oxygen levels in the brain over a sustained period of time or possibly by lowering a threshold or reserve capacity such that an otherwise silent cerebrovascular accident such as a small stroke or transient ischemic attack has a greater impact on subsequent cognition. Finally, all such relationships may also be related to other co-morbidities which could prompt both anaemia and cognitive impairment.

Although the data here are reflective of the literature there are many limitations to this investigation. The studies considered here were heterogeneous in that they looked at different populations, with one study looking at women only. Only one of the studies looked at different concentrations of haemoglobin in detail and took into account the possibility that the elderly may have different normal concentrations. Studies adjusted for confounders to different levels of detail. Definitions of dementia and cognitive decline varied between the studies. Despite many limitations, the studies did frequently use the standard WHO definition of anaemia.

## Conclusion

All of the above leads us to conclude that further studies to examine the effect of anaemia and haemoglobin on dementia and cognitive decline are needed. Such studies would need to be of large size and longitudinal design with robust cognitive assessment in the very elderly, in order to allow the evaluation of possible risks and benefits in treatment or prevention of dementia or amelioration of cognitive symptoms both physical and financial.

## Competing interests

The authors declare that they have no competing interests. Details of funding; none received and researchers therefore independent.

## Authors' contributions

All data gathering, analysis and writing was carried out by RP & LB. CB, NB and JW provided advice and critical appraisal of the analyses to be performed, methods and writing style. RPoulter reviewed and commented on the paper.

## Pre-publication history

The pre-publication history for this paper can be accessed here:


